# Supplementary material for: Profitability of Contrarian Strategies in the Chinese Stock Market
Source: PLoS One. 2015 Sep 14;10(9):e0137892. doi: 10.1371/journal.pone.0137892 (PMC4569377; doi:10.1371/journal.pone.0137892)
Supplement: S2 Fig — (PDF) [file pone.0137892.s002.pdf]

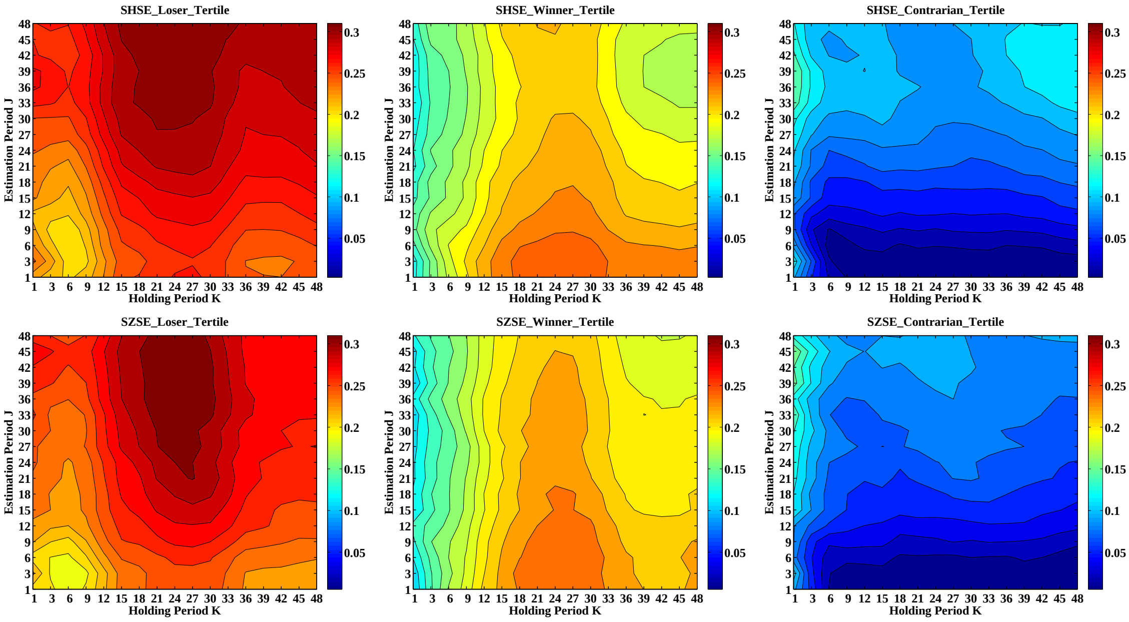

Figure S2: **Contour plots of the average annualized returns based on tertile grouping with varying estimation and holding horizons.** The left panel is for loser portfolios, the middle panel is for winner portfolios, and the right panel is for contrarian portfolios. The top panel is for the SHSE stocks and the bottom is for the SZSE stocks.
