# Supplementary material for: Profitability of Contrarian Strategies in the Chinese Stock Market
Source: PLoS One. 2015 Sep 14;10(9):e0137892. doi: 10.1371/journal.pone.0137892 (PMC4569377; doi:10.1371/journal.pone.0137892)
Supplement: S3 Fig — (PDF) [file pone.0137892.s003.pdf]

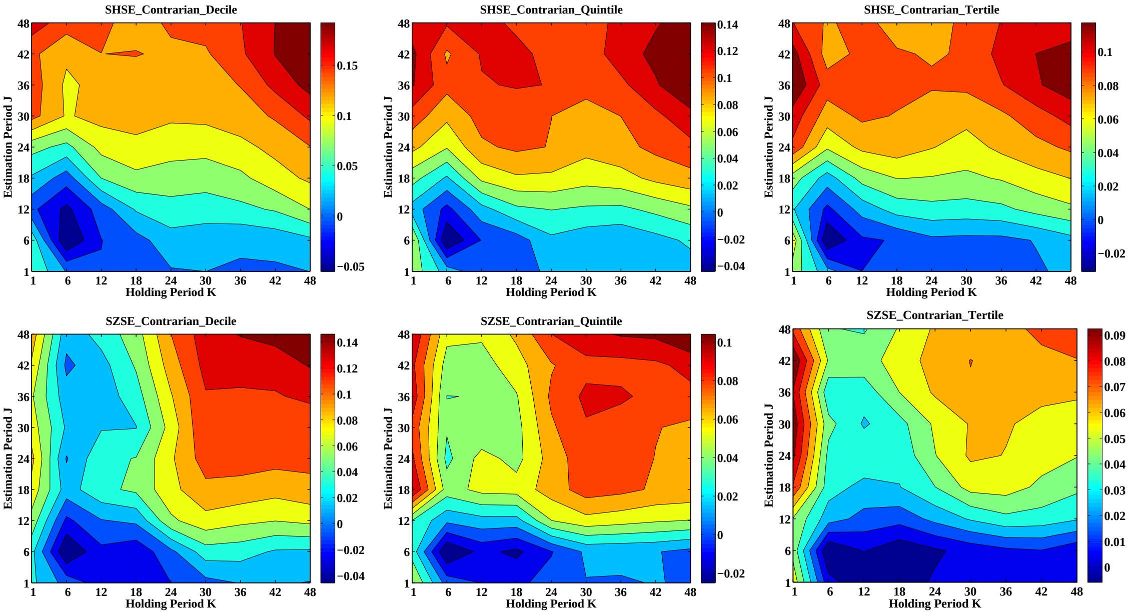

Figure S3: **Contour plots of the average annualized returns of contrarian portfolios in subperiod January 1997 to September 2007 with varying estimation and holding horizons.** The top panel is for SHSE stocks and the bottom panel for SZSE stocks. The panels from left to right correspond to the cases based on decile grouping, quintile grouping and tertile grouping.
