# Supplementary material for: Profitability of Contrarian Strategies in the Chinese Stock Market
Source: PLoS One. 2015 Sep 14;10(9):e0137892. doi: 10.1371/journal.pone.0137892 (PMC4569377; doi:10.1371/journal.pone.0137892)
Supplement: S5 Fig — (PDF) [file pone.0137892.s005.pdf]

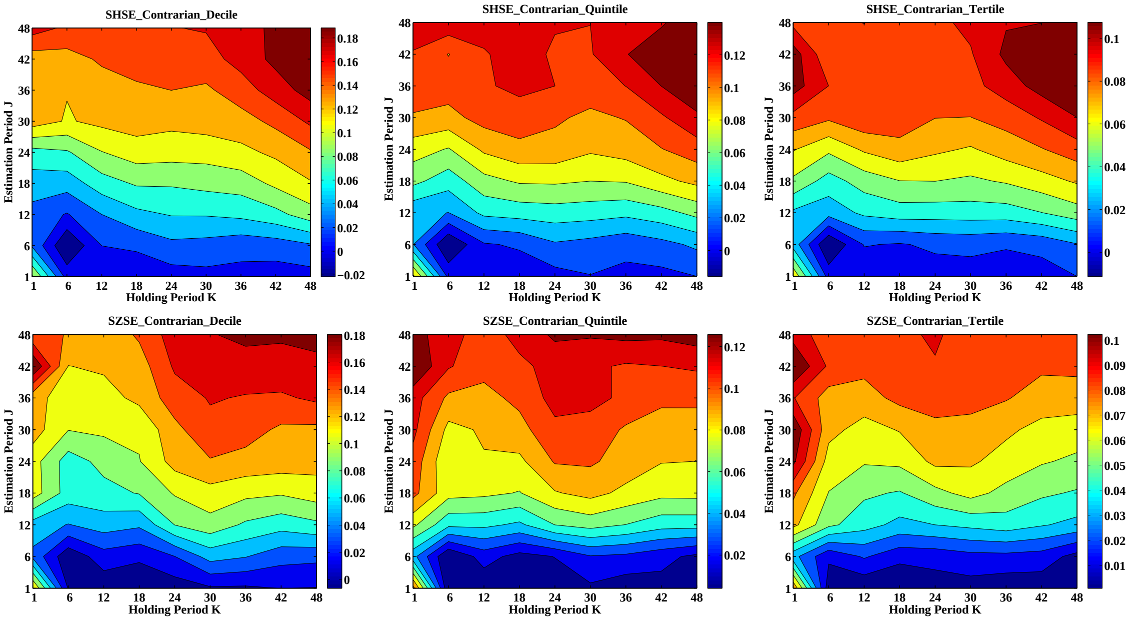

Figure S5: **Contour plots of the average annualized returns of contrarian portfolios with a one-month skipping between the estimation and holding periods.** The top panel is for SHSE stocks and the bottom panel for SZSE stocks. The sample period is from January 1997 to December 2012. The panels from left to right correspond to the cases based on decile grouping, quintile grouping and tertile grouping.
