# Supplementary material for: Profitability of Contrarian Strategies in the Chinese Stock Market
Source: PLoS One. 2015 Sep 14;10(9):e0137892. doi: 10.1371/journal.pone.0137892 (PMC4569377; doi:10.1371/journal.pone.0137892)
Supplement: S1 Table — (PDF) [file pone.0137892.s006.pdf]

Table S1. The annualized returns of the loser, winner, and contrarian portfolios on the SHSE formed based on J-month lagged returns and held for K months for the whole sample period 1997-2012.

	$K=1$		6		12		18		24		30		36		42		48	
J	Ret	t-stat	Ret	t-stat	Ret	t-stat	Ret	t-stat	Ret	t-stat	Ret	t-stat	Ret	t-stat	Ret	t-stat	Ret	t-stat
Panel A: Loser portfolio																		
1	0.228	2.33*	0.208	2.08*	0.231	2.35*	0.251	2.53*	0.260	2.66**	0.261	3.14**	0.242	3.79**	0.244	4.25**	0.249	4.17**
6	0.238	2.22*	0.194	1.95	0.232	2.33*	0.249	2.47*	0.264	2.66**	0.263	3.21**	0.248	3.89**	0.249	4.34**	0.255	4.23**
12	0.227	2.11*	0.207	2.04*	0.241	2.40*	0.262	2.55*	0.272	2.80**	0.274	3.35**	0.261	4.05**	0.259	4.47**	0.267	4.32**
18	0.245	2.28*	0.220	2.14*	0.258	2.50*	0.282	2.69**	0.291	2.95**	0.291	3.50**	0.277	4.18**	0.277	4.60**	0.285	4.45**
24	0.245	2.28*	0.235	2.28*	0.275	2.64**	0.298	2.81**	0.304	3.03**	0.299	3.58**	0.287	4.30**	0.288	4.70**	0.293	4.51**
30	0.271	2.47*	0.257	2.45*	0.289	2.77**	0.308	2.89**	0.313	3.11**	0.307	3.71**	0.293	4.49**	0.295	4.85**	0.305	4.57**
36	0.281	2.57*	0.273	2.56*	0.298	2.84**	0.318	2.96**	0.325	3.21**	0.318	3.85**	0.303	4.68**	0.308	4.99**	0.317	4.72**
42	0.285	2.58*	0.270	2.55*	0.300	2.86**	0.323	3.01**	0.325	3.27**	0.322	3.93**	0.315	4.78**	0.318	5.12**	0.322	4.81**
48	0.281	2.56*	0.279	2.64**	0.308	2.92**	0.325	3.05**	0.328	3.26**	0.324	3.90**	0.319	4.81**	0.319	5.20**	0.324	4.85**
Panel B: Winner portfolio																		
1	0.107	1.07	0.172	1.88	0.217	2.30*	0.236	2.39*	0.243	2.52*	0.239	2.94**	0.227	3.47**	0.227	3.94**	0.229	3.95**
6	0.129	1.35	0.186	1.99*	0.209	2.25*	0.230	2.34*	0.233	2.44*	0.235	2.77**	0.226	3.21**	0.222	3.63**	0.220	3.77**
12	0.147	1.52	0.168	1.86	0.190	2.09*	0.210	2.18*	0.216	2.29*	0.219	2.60*	0.210	3.01**	0.204	3.40**	0.203	3.57**
18	0.123	1.28	0.156	1.73	0.181	1.99*	0.200	2.08*	0.208	2.20*	0.211	2.53*	0.197	2.87**	0.188	3.30**	0.188	3.41**
24	0.119	1.24	0.155	1.70	0.175	1.93	0.191	2.04*	0.199	2.14*	0.202	2.44*	0.187	2.79**	0.180	3.22**	0.177	3.30**
30	0.120	1.24	0.146	1.62	0.170	1.89	0.188	1.99*	0.198	2.09*	0.199	2.39*	0.182	2.73**	0.174	3.16**	0.169	3.22**
36	0.118	1.21	0.145	1.60	0.169	1.86	0.185	1.96	0.195	2.07*	0.196	2.35*	0.177	2.69**	0.166	3.10**	0.160	3.19**
42	0.116	1.19	0.148	1.62	0.169	1.86	0.185	1.97	0.199	2.10*	0.197	2.41*	0.174	2.76**	0.169	3.19**	0.164	3.27**
48	0.120	1.22	0.139	1.53	0.168	1.85	0.190	2.02*	0.198	2.15*	0.195	2.49*	0.178	2.85**	0.173	3.26**	0.171	3.40**
Panel C: Contrarian portfolio																		
1	0.120	3.58**	0.037	2.17*	0.014	1.19	0.015	1.60	0.017	2.24*	0.022	2.43*	0.016	1.60	0.017	1.84	0.020	1.80
6	0.110	2.82**	0.008	0.26	0.023	0.92	0.018	1.01	0.031	1.75	0.028	1.47	0.022	1.00	0.027	1.29	0.035	1.70
12	0.080	1.95	0.040	1.15	0.051	1.75	0.052	2.32*	0.056	2.83**	0.055	2.62*	0.050	2.05*	0.055	2.20*	0.064	2.74**
18	0.122	2.76**	0.064	1.72	0.076	2.40*	0.082	3.24**	0.083	3.53**	0.080	3.30**	0.080	2.96**	0.088	3.30**	0.096	3.70**
24	0.126	2.82**	0.080	2.29*	0.099	3.05**	0.106	3.99**	0.106	4.43**	0.097	4.09**	0.100	3.67**	0.108	3.94**	0.116	4.08**
30	0.151	3.27**	0.111	2.97**	0.118	3.65**	0.120	4.72**	0.115	4.83**	0.108	4.26**	0.111	3.87**	0.121	4.10**	0.136	4.47**
36	0.164	3.44**	0.128	3.43**	0.129	4.13**	0.133	5.14**	0.130	5.41**	0.122	4.77**	0.126	4.49**	0.142	4.82**	0.156	5.08**
42	0.169	3.31**	0.122	3.27**	0.130	4.08**	0.137	5.46**	0.126	5.54**	0.125	5.31**	0.141	5.38**	0.148	5.18**	0.159	5.17**
48	0.161	3.24**	0.141	3.81**	0.140	4.62**	0.135	5.49**	0.129	6.06**	0.129	5.72**	0.141	5.83**	0.146	5.43**	0.153	4.87**

This table reports the average annualized returns and the corresponding t-statistics adjusted for heteroscedasticity and autocorrelation of the loser, winner and contrarian portfolios, which are formed by ranking the stocks based on their J-month lagged returns, adopting the quintile grouping, and holding for K months. The values of J and K for different strategies are indicated in the first collum and the first row respectively. The sample period is January 1997 to December 2012. The superscripts * and ** denote the significance at 5% and 1% levels, respectively.
