# Supplementary material for: Profitability of Contrarian Strategies in the Chinese Stock Market
Source: PLoS One. 2015 Sep 14;10(9):e0137892. doi: 10.1371/journal.pone.0137892 (PMC4569377; doi:10.1371/journal.pone.0137892)
Supplement: S2 Table — (PDF) [file pone.0137892.s007.pdf]

Table S2. The annualized returns of the loser, winner, and contrarian portfolios on the SZSE formed based on J-month lagged returns and held for K months for the whole sample period 1997-2012.

	$K=1$		6		12		18		24		30		36		42		48	
J	Ret	t-stat	Ret	t-stat	Ret	t-stat	Ret	t-stat	Ret	t-stat	Ret	t-stat	Ret	t-stat	Ret	t-stat	Ret	t-stat
Panel A: Loser portfolio																		
1	0.203	2.13*	0.184	1.89	0.213	2.18*	0.237	2.43*	0.250	2.59*	0.253	3.02**	0.232	3.61**	0.230	4.18**	0.230	4.03**
6	0.206	2.00*	0.189	1.90	0.224	2.27*	0.241	2.46*	0.255	2.61*	0.259	3.07**	0.241	3.66**	0.242	4.21**	0.237	4.00**
12	0.242	2.16*	0.221	2.15*	0.250	2.52*	0.266	2.70**	0.290	2.82**	0.292	3.25**	0.273	3.91**	0.267	4.44**	0.265	4.22**
18	0.260	2.36*	0.239	2.27*	0.266	2.59*	0.290	2.78**	0.312	2.88**	0.314	3.34**	0.288	4.05**	0.278	4.56**	0.279	4.30**
24	0.259	2.36*	0.237	2.25*	0.271	2.59*	0.295	2.78**	0.321	2.93**	0.315	3.41**	0.290	4.13**	0.282	4.61**	0.282	4.34**
30	0.265	2.44*	0.251	2.38*	0.280	2.70**	0.311	2.90**	0.330	3.07**	0.322	3.54**	0.295	4.23**	0.288	4.71**	0.284	4.37**
36	0.277	2.52*	0.266	2.49*	0.295	2.77**	0.317	2.96**	0.338	3.16**	0.332	3.68**	0.308	4.42**	0.297	4.86**	0.297	4.56**
42	0.302	2.66**	0.276	2.56*	0.297	2.78**	0.322	2.98**	0.338	3.17**	0.328	3.70**	0.301	4.48**	0.295	5.00**	0.297	4.58**
48	0.292	2.61*	0.280	2.54*	0.295	2.75**	0.324	2.97**	0.346	3.25**	0.330	3.84**	0.308	4.69**	0.304	5.11**	0.308	4.68**
Panel B: Winner portfolio																		
1	0.094	0.92	0.160	1.73	0.199	2.11*	0.224	2.22*	0.236	2.42*	0.234	2.87**	0.217	3.47**	0.213	3.90**	0.216	3.80**
6	0.118	1.24	0.170	1.79	0.201	2.08*	0.228	2.18*	0.236	2.37*	0.230	2.73**	0.217	3.28**	0.219	3.62**	0.224	3.59**
12	0.144	1.48	0.160	1.69	0.193	1.93	0.217	2.07*	0.224	2.25*	0.222	2.61*	0.209	3.06**	0.208	3.36**	0.209	3.42**
18	0.107	1.10	0.147	1.55	0.180	1.83	0.207	1.96	0.222	2.13*	0.217	2.45*	0.197	2.85**	0.195	3.21**	0.198	3.30**
24	0.108	1.10	0.149	1.55	0.177	1.83	0.208	1.92	0.217	2.03*	0.209	2.35*	0.190	2.76**	0.190	3.18**	0.192	3.24**
30	0.121	1.21	0.152	1.58	0.187	1.86	0.213	1.93	0.218	2.01*	0.208	2.33*	0.191	2.78**	0.190	3.15**	0.193	3.18**
36	0.125	1.25	0.161	1.65	0.194	1.90	0.213	1.95	0.218	2.02*	0.211	2.36*	0.192	2.81**	0.189	3.13**	0.190	3.18**
42	0.134	1.32	0.156	1.59	0.189	1.83	0.209	1.90	0.218	2.00*	0.208	2.30*	0.186	2.66**	0.180	2.99**	0.183	3.13**
48	0.121	1.16	0.150	1.53	0.180	1.76	0.204	1.85	0.212	1.93	0.198	2.19*	0.175	2.52*	0.174	2.94**	0.175	3.09**
Panel C: Contrarian portfolio																		
1	0.109	3.33**	0.025	1.78	0.014	1.18	0.013	0.84	0.014	1.24	0.018	2.05*	0.015	1.77	0.017	1.91	0.014	1.29
6	0.088	2.36*	0.020	0.61	0.023	0.90	0.013	0.51	0.018	1.04	0.029	1.84	0.024	1.39	0.022	1.01	0.014	0.60
12	0.098	2.07*	0.061	1.74	0.057	1.77	0.049	1.81	0.067	3.23**	0.070	3.27**	0.065	2.41*	0.059	1.94	0.056	1.87
18	0.154	3.36**	0.092	2.32*	0.086	2.58*	0.083	2.97**	0.090	3.59**	0.097	3.38**	0.090	2.84**	0.083	2.52*	0.082	2.69**
24	0.151	2.86**	0.088	2.12*	0.094	2.76**	0.087	2.78**	0.104	3.56**	0.107	3.62**	0.100	3.03**	0.092	2.85**	0.090	2.78**
30	0.144	2.68**	0.099	2.38*	0.093	2.54*	0.098	2.96**	0.113	3.56**	0.114	3.80**	0.105	3.31**	0.097	3.03**	0.091	2.75**
36	0.151	2.73**	0.104	2.32*	0.101	2.50*	0.104	3.03**	0.120	3.75**	0.121	4.02**	0.116	3.66**	0.108	3.17**	0.107	3.14**
42	0.168	3.02**	0.121	2.73**	0.109	2.55*	0.112	3.28**	0.120	3.90**	0.120	4.04**	0.115	3.52**	0.115	3.44**	0.114	3.62**
48	0.172	2.98**	0.130	2.76**	0.114	2.70**	0.120	3.56**	0.134	4.21**	0.132	4.10**	0.133	4.00**	0.130	3.95**	0.133	4.60**

This table reports the average annualized returns and the corresponding t-statistics adjusted for heteroscedasticity and autocorrelation of the loser, winner and contrarian portfolios, which are formed by ranking the stocks based on their J-month lagged returns, adopting the quintile grouping, and holding for K months. The values of J and K for different strategies are indicated in the first collum and the first row respectively. The sample period is January 1997 to December 2012. The superscripts * and ** denote the significance at 5% and 1% levels, respectively.
