# Supplementary material for: Profitability of Contrarian Strategies in the Chinese Stock Market
Source: PLoS One. 2015 Sep 14;10(9):e0137892. doi: 10.1371/journal.pone.0137892 (PMC4569377; doi:10.1371/journal.pone.0137892)
Supplement: S3 Table — (PDF) [file pone.0137892.s008.pdf]

Table S3. The annualized returns of the loser, winner, and contrarian portfolios on the SHSE formed based on J-month lagged returns and held for K months for the whole sample period 1997-2012.

	$K = 1$		6		12		18		24		30		36		42		48	
J	Ret	t-stat	Ret	t-stat	Ret	t-stat	Ret	t-stat	Ret	t-stat	Ret	t-stat	Ret	t-stat	Ret	t-stat	Ret	t-stat
Panel A: Loser portfolio																		
1	0.227	2.30*	0.210	2.11*	0.233	2.39*	0.254	2.55*	0.264	2.67**	0.261	3.16**	0.243	3.81**	0.243	4.28**	0.248	4.20**
6	0.234	2.18*	0.203	2.04*	0.237	2.40*	0.256	2.53*	0.264	2.70**	0.264	3.25**	0.247	3.95**	0.247	4.40**	0.253	4.29**
12	0.220	2.06*	0.214	2.11*	0.246	2.47*	0.269	2.63**	0.278	2.85**	0.276	3.40**	0.259	4.10**	0.260	4.55**	0.267	4.38**
18	0.238	2.25*	0.224	2.21*	0.257	2.55*	0.281	2.73**	0.288	2.94**	0.288	3.48**	0.271	4.19**	0.271	4.61**	0.278	4.45**
24	0.245	2.28*	0.236	2.29*	0.269	2.64**	0.294	2.80**	0.300	3.00**	0.296	3.57**	0.281	4.28**	0.281	4.68**	0.286	4.51**
30	0.253	2.36*	0.252	2.42*	0.282	2.75**	0.301	2.88**	0.305	3.07**	0.301	3.65**	0.284	4.42**	0.285	4.84**	0.291	4.64**
36	0.275	2.54*	0.263	2.52*	0.287	2.80**	0.304	2.91**	0.310	3.13**	0.305	3.74**	0.290	4.56**	0.293	4.95**	0.299	4.75**
42	0.264	2.45*	0.257	2.48*	0.284	2.79**	0.305	2.94**	0.310	3.17**	0.307	3.82**	0.296	4.64**	0.297	5.05**	0.298	4.83**
48	0.262	2.43*	0.264	2.53*	0.293	2.84**	0.307	2.98**	0.313	3.21**	0.312	3.83**	0.300	4.63**	0.296	5.10**	0.298	4.88**
Panel B: Winner portfolio																		
1	0.126	1.24	0.183	1.96	0.224	2.37*	0.245	2.47*	0.252	2.62*	0.248	3.05**	0.233	3.60**	0.231	4.08**	0.232	4.08**
6	0.140	1.45	0.193	2.03*	0.217	2.33*	0.241	2.43*	0.247	2.55*	0.245	2.91**	0.231	3.40**	0.229	3.87**	0.229	3.93**
12	0.148	1.52	0.177	1.90	0.204	2.20*	0.226	2.31*	0.233	2.41*	0.232	2.78**	0.216	3.28**	0.212	3.70**	0.212	3.76**
18	0.138	1.42	0.170	1.84	0.201	2.17*	0.219	2.25*	0.228	2.37*	0.227	2.76**	0.210	3.19**	0.204	3.64**	0.204	3.67**
24	0.131	1.35	0.166	1.79	0.193	2.09*	0.212	2.21*	0.222	2.34*	0.223	2.69**	0.202	3.13**	0.197	3.56**	0.196	3.57**
30	0.123	1.27	0.157	1.72	0.185	2.03*	0.206	2.16*	0.218	2.29*	0.215	2.63**	0.195	3.05**	0.187	3.47**	0.183	3.47**
36	0.124	1.27	0.155	1.69	0.184	2.01*	0.204	2.14*	0.214	2.26*	0.208	2.59*	0.186	2.99**	0.177	3.40**	0.175	3.43**
42	0.123	1.26	0.159	1.72	0.186	2.02*	0.208	2.16*	0.216	2.28*	0.210	2.61*	0.185	2.99**	0.179	3.43**	0.178	3.46**
48	0.136	1.37	0.163	1.75	0.192	2.05*	0.213	2.20*	0.218	2.33*	0.211	2.67**	0.191	3.10**	0.185	3.53**	0.183	3.58**
Panel C: Contrarian portfolio																		
1	0.101	3.62**	0.028	2.14*	0.009	0.95	0.009	1.29	0.012	1.89	0.013	1.69	0.010	1.33	0.012	1.60	0.016	1.89
6	0.094	3.07**	0.010	0.41	0.021	1.04	0.015	1.02	0.017	1.25	0.019	1.25	0.016	0.98	0.018	1.12	0.025	1.59
12	0.073	2.29*	0.037	1.36	0.042	1.82	0.043	2.40*	0.045	2.82**	0.045	2.60*	0.043	2.26*	0.048	2.46*	0.054	3.05**
18	0.101	2.90**	0.054	1.83	0.056	2.21*	0.061	3.02**	0.061	3.21**	0.061	3.10**	0.061	2.80**	0.067	3.08**	0.073	3.55**
24	0.114	3.23**	0.071	2.41*	0.077	2.93**	0.082	3.83**	0.078	4.01**	0.073	3.67**	0.079	3.58**	0.084	3.70**	0.091	3.88**
30	0.129	3.61**	0.095	3.17**	0.097	3.69**	0.095	4.56**	0.087	4.60**	0.086	4.28**	0.089	4.11**	0.097	4.26**	0.108	4.51**
36	0.151	4.18**	0.108	3.61**	0.103	4.03**	0.100	4.96**	0.096	5.38**	0.097	5.14**	0.104	4.82**	0.116	5.05**	0.124	5.09**
42	0.141	3.77**	0.097	3.34**	0.099	3.90**	0.097	5.06**	0.093	5.32**	0.097	5.26**	0.112	5.35**	0.118	5.54**	0.120	5.32**
48	0.127	3.36**	0.101	3.34**	0.102	3.92**	0.094	4.77**	0.096	5.39**	0.101	5.43**	0.110	6.02**	0.111	5.84**	0.114	5.35**

This table reports the average annualized returns and the corresponding t-statistics adjusted for heteroscedasticity and autocorrelation of the loser, winner and contrarian portfolios, which are formed by ranking the stocks based on their J-month lagged returns, adopting the tertile grouping, and holding for K months. The values of J and K for different strategies are indicated in the first collum and the first row respectively. The sample period is January 1997 to December 2012. The superscripts * and ** denote the significance at 5% and 1% levels, respectively.
