# Supplementary material for: Profitability of Contrarian Strategies in the Chinese Stock Market
Source: PLoS One. 2015 Sep 14;10(9):e0137892. doi: 10.1371/journal.pone.0137892 (PMC4569377; doi:10.1371/journal.pone.0137892)
Supplement: S4 Table — (PDF) [file pone.0137892.s009.pdf]

Table S4. The annualized returns of the loser, winner, and contrarian portfolios on the SZSE formed based on J-month lagged returns and held for K months for the whole sample period 1997-2012.

	$K=1$		6		12		18		24		30		36		42		48	
J	Ret	t-stat	Ret	t-stat	Ret	t-stat	Ret	t-stat	Ret	t-stat	Ret	t-stat	Ret	t-stat	Ret	t-stat	Ret	t-stat
Panel A: Loser portfolio																		
1	0.207	2.12*	0.193	1.95	0.222	2.26*	0.246	2.47*	0.258	2.59*	0.255	3.04**	0.234	3.70**	0.230	4.24**	0.232	4.06**
6	0.207	1.98*	0.198	1.96	0.233	2.33*	0.252	2.50*	0.262	2.65**	0.261	3.14**	0.242	3.78**	0.240	4.33**	0.236	4.10**
12	0.232	2.08*	0.224	2.13*	0.250	2.48*	0.268	2.63**	0.282	2.78**	0.282	3.23**	0.264	3.94**	0.257	4.47**	0.254	4.24**
18	0.245	2.24*	0.233	2.23*	0.256	2.52*	0.281	2.67**	0.296	2.82**	0.296	3.31**	0.272	4.06**	0.263	4.54**	0.262	4.29**
24	0.250	2.29*	0.234	2.23*	0.261	2.53*	0.287	2.70**	0.307	2.90**	0.299	3.43**	0.274	4.17**	0.268	4.61**	0.269	4.37**
30	0.254	2.32*	0.241	2.29*	0.269	2.60*	0.297	2.75**	0.312	2.97**	0.302	3.51**	0.279	4.23**	0.272	4.69**	0.270	4.41**
36	0.255	2.35*	0.248	2.33*	0.277	2.63**	0.305	2.82**	0.317	3.05**	0.311	3.59**	0.286	4.34**	0.276	4.78**	0.276	4.51**
42	0.270	2.43*	0.259	2.41*	0.280	2.67**	0.305	2.85**	0.319	3.08**	0.309	3.64**	0.282	4.41**	0.275	4.89**	0.276	4.51**
48	0.264	2.38*	0.255	2.34*	0.277	2.61*	0.302	2.83**	0.316	3.11**	0.304	3.68**	0.281	4.49**	0.277	4.92**	0.280	4.54**
Panel B: Winner portfolio																		
1	0.110	1.06	0.173	1.82	0.214	2.22*	0.236	2.33*	0.247	2.52*	0.242	2.96**	0.224	3.60**	0.221	4.03**	0.224	3.92**
6	0.123	1.27	0.172	1.82	0.206	2.16*	0.234	2.30*	0.245	2.47*	0.241	2.83**	0.224	3.42**	0.220	3.86**	0.226	3.79**
12	0.139	1.41	0.163	1.71	0.202	2.05*	0.228	2.21*	0.239	2.36*	0.236	2.77**	0.217	3.31**	0.213	3.68**	0.216	3.66**
18	0.124	1.26	0.159	1.65	0.197	2.01*	0.224	2.15*	0.236	2.31*	0.229	2.70**	0.209	3.20**	0.207	3.62**	0.211	3.59**
24	0.120	1.23	0.156	1.62	0.191	1.96	0.222	2.11*	0.230	2.23*	0.220	2.58*	0.202	3.09**	0.202	3.53**	0.206	3.52**
30	0.117	1.19	0.153	1.60	0.197	1.99*	0.221	2.11*	0.229	2.21*	0.221	2.57*	0.202	3.08**	0.199	3.49**	0.202	3.51**
36	0.127	1.27	0.166	1.69	0.198	2.00*	0.217	2.06*	0.227	2.19*	0.220	2.54*	0.200	3.02**	0.197	3.41**	0.198	3.46**
42	0.120	1.20	0.159	1.62	0.191	1.91	0.214	2.02*	0.224	2.14*	0.216	2.47*	0.196	2.89**	0.189	3.29**	0.192	3.38**
48	0.133	1.31	0.159	1.62	0.190	1.89	0.210	1.99*	0.220	2.08*	0.213	2.39*	0.190	2.78**	0.184	3.22**	0.186	3.34**
Panel C: Contrarian portfolio																		
1	0.097	3.71**	0.020	1.75	0.008	0.92	0.010	0.87	0.012	1.36	0.012	1.72	0.010	1.51	0.009	1.38	0.008	0.91
6	0.084	2.89**	0.026	1.09	0.027	1.38	0.018	0.90	0.018	1.19	0.020	1.40	0.019	1.25	0.020	1.18	0.011	0.57
12	0.093	2.49*	0.061	2.16*	0.049	1.94	0.040	2.05*	0.043	2.79**	0.045	2.78**	0.047	2.32*	0.044	1.92	0.038	1.63
18	0.121	3.32**	0.074	2.37*	0.058	2.22*	0.057	2.72**	0.060	3.18**	0.067	3.08**	0.064	2.62*	0.056	2.27*	0.051	2.11*
24	0.130	3.13**	0.078	2.37*	0.071	2.65**	0.066	2.80**	0.077	3.32**	0.079	3.24**	0.072	2.81**	0.066	2.51*	0.063	2.32*
30	0.137	3.23**	0.089	2.73**	0.072	2.50*	0.075	3.12**	0.083	3.54**	0.081	3.43**	0.077	3.03**	0.073	2.71**	0.068	2.50*
36	0.128	3.02**	0.083	2.47*	0.079	2.65**	0.088	3.47**	0.090	3.72**	0.090	3.64**	0.086	3.17**	0.079	2.73**	0.078	2.79**
42	0.150	3.57**	0.100	3.13**	0.089	2.96**	0.091	3.77**	0.095	4.10**	0.093	3.81**	0.087	3.16**	0.086	3.09**	0.084	3.26**
48	0.131	2.84**	0.095	2.76**	0.087	2.70**	0.092	3.56**	0.096	3.75**	0.091	3.37**	0.090	3.25**	0.093	3.61**	0.094	4.12**

This table reports the average annualized returns and the corresponding t-statistics adjusted for heteroscedasticity and autocorrelation of the loser, winner and contrarian portfolios, which are formed by ranking the stocks based on their J-month lagged returns, adopting the tertile grouping, and holding for K months. The values of J and K for different strategies are indicated in the first collum and the first row respectively. The sample period is January 1997 to December 2012. The superscripts * and ** denote the significance at 5% and 1% levels, respectively.
