# Supplementary material for: Profitability of Contrarian Strategies in the Chinese Stock Market
Source: PLoS One. 2015 Sep 14;10(9):e0137892. doi: 10.1371/journal.pone.0137892 (PMC4569377; doi:10.1371/journal.pone.0137892)
Supplement: S7 Table — (PDF) [file pone.0137892.s012.pdf]

Table S7. The return difference of winner portfolios formed based on different grouping ways of the SHSE stocks.

	$K=1$		6		12		18		24		30		36		42		48	
J	ΔR	t-stat	ΔR	t-stat	ΔR	t-stat	ΔR	t-stat	ΔR	t-stat	ΔR	t-stat	ΔR	t-stat	ΔR	t-stat	ΔR	t-stat
Panel A: $G_5 - G_3$																		
1	-0.018	-2.40*	-0.011	-3.37**	-0.008	-2.85**	-0.009	-3.84**	-0.009	-3.88**	-0.009	-3.71**	-0.006	-2.48*	-0.004	-1.66	-0.003	-1.06
6	-0.011	-1.33	-0.007	-2.07*	-0.008	-2.68**	-0.010	-4.30**	-0.014	-6.00**	-0.010	-3.90**	-0.005	-1.79	-0.007	-2.81**	-0.009	-3.98**
12	-0.000	-0.03	-0.009	-2.28*	-0.014	-4.43**	-0.016	-5.21**	-0.017	-6.27**	-0.013	-4.61**	-0.006	-1.81	-0.008	-2.66**	-0.010	-3.79**
18	-0.015	-1.69	-0.014	-3.78**	-0.019	-6.29**	-0.020	-7.57**	-0.020	-7.92**	-0.017	-6.20**	-0.013	-4.66**	-0.016	-7.28**	-0.016	-7.11**
24	-0.012	-1.38	-0.011	-2.71**	-0.017	-5.50**	-0.021	-8.39**	-0.024	-8.80**	-0.021	-8.79**	-0.015	-5.76**	-0.018	-7.86**	-0.018	-8.96**
30	-0.004	-0.38	-0.011	-2.56*	-0.015	-5.09**	-0.018	-6.84**	-0.020	-7.22**	-0.016	-5.68**	-0.013	-4.26**	-0.014	-6.07**	-0.014	-6.59**
36	-0.006	-0.70	-0.010	-2.67**	-0.015	-4.62**	-0.019	-6.86**	-0.018	-6.32**	-0.013	-4.13**	-0.009	-3.16**	-0.012	-4.95**	-0.015	-7.30**
42	-0.007	-0.73	-0.012	-2.92**	-0.016	-5.21**	-0.022	-7.95**	-0.017	-6.75**	-0.013	-4.72**	-0.010	-4.30**	-0.009	-3.26**	-0.014	-4.99**
48	-0.016	-1.33	-0.024	-4.96**	-0.024	-7.29**	-0.023	-7.41**	-0.020	-7.30**	-0.016	-5.15**	-0.013	-4.65**	-0.012	-3.62**	-0.012	-3.46**
Panel B: $G_{10} - G_5$																		
1	-0.022	-1.79	-0.007	-1.64	-0.006	-1.44	-0.005	-1.00	-0.006	-1.67	-0.002	-0.54	-0.001	-0.31	0.002	0.55	0.001	0.16
6	-0.015	-1.30	-0.010	-2.07*	-0.017	-4.31**	-0.015	-4.24**	-0.014	-4.60**	-0.017	-5.31**	-0.013	-3.97**	-0.007	-1.97	-0.008	-2.23*
12	-0.007	-0.66	-0.003	-0.55	-0.005	-1.27	-0.009	-2.60*	-0.013	-3.78**	-0.009	-2.64**	-0.007	-1.52	-0.007	-1.45	-0.016	-4.31**
18	-0.005	-0.38	-0.009	-1.50	-0.012	-2.77**	-0.015	-3.98**	-0.015	-4.08**	-0.015	-3.64**	-0.009	-2.09*	-0.012	-3.11**	-0.016	-4.87**
24	-0.004	-0.35	-0.013	-2.21*	-0.012	-2.83**	-0.014	-4.10**	-0.009	-2.31*	-0.012	-3.05**	-0.010	-2.92**	-0.010	-2.87**	-0.010	-3.43**
30	-0.028	-2.29*	-0.015	-3.09**	-0.015	-3.63**	-0.013	-3.38**	-0.010	-2.81**	-0.009	-2.49*	-0.007	-2.06*	-0.008	-2.30*	-0.010	-3.23**
36	-0.014	-1.02	-0.006	-1.00	-0.006	-1.35	-0.006	-1.53	-0.005	-1.17	-0.004	-0.90	-0.005	-1.63	-0.008	-2.70**	-0.013	-4.62**
42	-0.006	-0.48	-0.016	-2.29*	-0.011	-2.41*	-0.004	-0.79	-0.005	-0.97	-0.005	-1.03	-0.008	-1.96	-0.017	-4.51**	-0.021	-5.63**
48	-0.025	-1.61	-0.021	-3.08**	-0.010	-2.09*	-0.006	-1.19	-0.008	-1.55	-0.009	-1.79	-0.008	-1.70	-0.019	-4.27**	-0.025	-4.75**
Panel C: $G_{10} - G_3$																		
1	-0.040	-2.45*	-0.019	-3.08**	-0.014	-2.54*	-0.014	-2.53*	-0.015	-3.32**	-0.011	-2.07*	-0.007	-1.42	-0.002	-0.55	-0.002	-0.49
6	-0.026	-1.51	-0.018	-2.50*	-0.025	-4.45**	-0.025	-5.25**	-0.028	-6.69**	-0.027	-6.22**	-0.017	-3.83**	-0.014	-2.73**	-0.017	-3.50**
12	-0.007	-0.44	-0.012	-1.51	-0.019	-3.15**	-0.025	-4.72**	-0.029	-6.25**	-0.022	-4.25**	-0.013	-1.87	-0.015	-2.15*	-0.026	-5.19**
18	-0.019	-1.08	-0.023	-2.79**	-0.031	-4.92**	-0.035	-6.74**	-0.035	-7.10**	-0.031	-5.46**	-0.022	-3.42**	-0.028	-5.49**	-0.032	-7.42**
24	-0.016	-0.90	-0.023	-2.84**	-0.029	-4.76**	-0.035	-7.20**	-0.032	-6.87**	-0.032	-7.09**	-0.025	-4.98**	-0.027	-5.81**	-0.029	-6.87**
30	-0.031	-1.64	-0.026	-3.33**	-0.029	-4.87**	-0.032	-5.98**	-0.030	-5.86**	-0.025	-4.71**	-0.019	-3.53**	-0.021	-4.63**	-0.024	-5.66**
36	-0.021	-1.03	-0.016	-1.86	-0.021	-3.07**	-0.025	-4.46**	-0.023	-3.85**	-0.016	-2.49*	-0.014	-2.65**	-0.019	-4.79**	-0.028	-6.90**
42	-0.013	-0.69	-0.027	-2.85**	-0.028	-4.34**	-0.026	-4.57**	-0.022	-3.45**	-0.018	-2.87**	-0.018	-3.56**	-0.026	-6.00**	-0.035	-8.04**
48	-0.041	-2.10*	-0.045	-4.40**	-0.034	-5.14**	-0.029	-4.99**	-0.028	-4.63**	-0.025	-4.63**	-0.021	-3.58**	-0.031	-6.31**	-0.037	-8.01**

This table reports the differences of the average annualized returns and the corresponding t-statistics of two winner strategies that are different only in the grouping methods for SHSE stocks. The three panels are for the loser, winner and contrarian portfolios, respectively. In the first row, G_3, G_5 and G_{10} stand for tertile, quintile and decile groupings. The sample period is January 1997 to December 2012. The superscripts * and ** denote the significance at 5% and 1% levels, respectively.
